# Retraction Note: A thematic analysis of system wide learning from first wave Covid-19 in the east of England

**DOI:** 10.1186/s12913-022-08263-0

**Published:** 2022-07-05

**Authors:** Carolyn Jackson, Kim Manley, Jonathan Webster, Sally Hardy

**Affiliations:** 1 Present Address: School of Health Sciences, University of East Anglia, Norwich Research Park, Norwich, NR7 4TJ UK; 2 Faculty of Medicine and Health Sciences, University of East Anglia, Norwich Research Park, Norwich, NR7 4TJ UK

**Retraction Note: BMC Health Services Research 22, 552 (2022)**

**https://doi.org/10.1186/s12913-022-07797-7**

The Editor has retracted this article because it contains material that overlaps with the following articles [1, 2]. All authors agree to this retraction.
